# Preteens social media use: Parents' and children's perceptions of what mediation approaches are used and why

**DOI:** 10.1111/bjdp.12552

**Published:** 2025-02-25

**Authors:** Sarah E. Rose, Louise R. Middling

**Affiliations:** ^1^ University of Satffordshire Stoke‐on‐Trent UK

**Keywords:** mediation, paired interviews, preteens, social media

## Abstract

Many preteens are using social media, despite most platforms having an age requirement of 13‐year‐old. Little is known about how these young users and their parents balance out the opportunities and potential risks of their social media use. To address this gap in our understanding we interviewed nine children (aged 9–12) together with one of their parents to address two research questions: (1) ‘What strategies are used to mediate social media use among preteens?’ (2) ‘What are parents and children's reflections of why different strategies are chosen?’ The findings identify a broader range of mediation strategies than previously discussed in the literature, challenge research suggesting that parents have a typical mediation approach and give new insight into children's role in co‐constructing how the mediation strategies are used. This has implications for future policies, interventions and research into the effectiveness of different mediation approaches.

## Graphical abstract

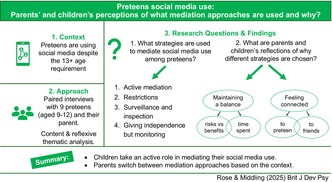

**Statement of Contribution**

**What is already known on this subject?**

- Despite social media platforms typically requiring users to be at least 13 years of age, many younger children are regular users.
- Parents initiate various mediation strategies to balance the benefits and risks associated with social media use.

**What does the present study add?**

- Children take an active role in mediating their own social media use.
- Parents used a wider range of mediation approaches than reflected in previous findings.
- The current findings challenge the idea that parents have a typical mediation approach, instead parents use multiple approaches interchangeably depending on context.

## INTRODUCTION

Social media is prevalent in everyday life, growing in popularity with approximately 4.95 billion active users worldwide (Statista Search Department, 2023). Social media platforms are diverse, and definitions have varied over time, but there is consensus that a defining feature of social media platforms is the opportunities that they provide for people to virtually connect and interact (Aichner et al., 2021). As children approach adolescence, they become increasingly aware of social media and start to value the potential benefits these platforms provide, such as facilitating new and exciting ways to pursue friendships and pursuing new hobbies and interests (Barbovschi et al., 2015; Hayes et al., 2021).

Despite social media platforms typically requiring users to be at least 13 years of age, many younger children are regular users. For example, 38% of 8‐ to 12‐year‐olds in America have used social media (Rideout, 2021), and in the United Kingdom, the number of 8‐ to 10‐year‐olds with a social media profile has increased threefold from 21% in 2019 to 63% in 2023 (Ofcom, 2019, 2023) with YouTube, WhatsApp and TikTok being the most popular platforms used by 8‐ to 11‐year‐olds (Ofcom, 2024). It seems that preteens using social media sites is quickly becoming an established normative behaviour within society (Jennings & Caplovitz, 2022).

Although children want to explore social media from an increasingly early age, research has found that they often lack the skills and understanding needed to navigate these platforms successfully (Livingstone et al., 2013; Pescott, 2020). For example, although 12‐year‐olds have difficulty understanding how the internet functions, they trust the information available on the internet (Brodsky et al., 2021). Similarly, Girouard‐Hallam and Danovitch (2025) found that the children in their sample (including the oldest age group of 9‐ to 10‐year‐olds) perceived Google as a more reliable and faster source of answers to factual questions about the past, present and future than a person. Moreover, children's positivity bias (Boseovski, 2010) may increase their vulnerability as Gelman et al. (2018) found that 10‐year‐olds are not concerned about a stranger digitally tracking their location. This contrasts with the privacy concerns and distrust about digital tracking expressed by adults and children over the age of 13 (Gelman et al., 2021). Likewise, Starks and Reich (2024) investigated preteens' (8‐to‐11‐year‐olds) understanding of digital privacy and found that although all children in their sample used social media, few had strategies to manage personal information and its privacy. Therefore, although many preteens, commonly defined as 8‐ to 12‐year‐olds, are users of social media, the evidence suggests these users are still developing the ability to think critically and to regulate their behaviour online. This lack of understanding and experience can leave them vulnerable to cyberbullying, grooming, glorification of self‐harm, radicalisation and exposure to adult content (Annansingh & Veli, 2016; Kopecký et al., 2020). This presents unique challenges for parents in trying to balance protecting their preteen from the potential risks of being online and wanting them to fit in with their peers (Livingstone & Smith, 2014; Smahelova et al., 2017) and experience potential positive outcomes of increased social support (Cole et al., 2017), friendships (Barbovschi et al., 2015) and sense of belonging (Smith et al., 2021).

Despite the age limits on social media platforms, many parents choose to facilitate their preteens use by creating an account for them (Jennings & Caplovitz, 2022; Ofcom, 2024). However, parents also initiate various mediation strategies to balance the benefits and risks associated with social media use (Hefner et al., 2019; Ho et al., 2020; McDonald‐Brown et al., 2017). Yet relatively little is known about parents' and preteens' perceptions and experiences of these mediation strategies, or their perceptions of what factors may influence which strategies are used. Therefore, this research paper aims to collect data from preteens and their parents to ascertain what strategies are used and their reflections on what influences strategy choice.

### Parental mediation theory

The concept of parents using mediation strategies to help mitigate the adverse effects of wider digital media on children is not new. Mediation strategies for television include limiting time spent or content watched (*restrictive mediation*), explaining potential risks and making suggestions to help mitigate them (*active mediation*) and watching content together (*co‐viewing*) (Nathanson, 2001). For mediation of internet, parents have relied primarily on active mediation and restrictions (Livingstone et al., 2017) although Nikken and Jansz (2014) also found evidence from their survey of parents of Dutch children aged 2‐ to 12‐year‐olds for strategies of *co‐use*, *supervision and technical safety guidelines*. A recent meta‐analysis suggests that parental mediation strategies, especially restrictive mediation, are effective in reducing potential harm from digital media (Chen & Shi, 2019).

Another approach used by some parents to mediate their preteens internet use is digital parental controls. These software solutions enable a user to control some, or all, of the functions of a device or service. Traditionally, many of these tools have focused on monitoring usage and safety restrictions (e.g. limiting time spent or access to certain content); however, some now have nudging features (e.g. educational messages) designed to encourage users to develop healthy internet use (Bertrandias et al., 2023). However, the use of digital parental controls is not widespread, with recent data from a European survey of parents of 9‐ to 16‐year‐olds suggesting that use varied from about 11% in Lithuania to approximately a third of parents in Poland, Norway and Spain (Smahel et al., 2020). However, it has been found that parents of preteens are more likely to use digital parental controls compared to parents of adolescents (Stoilova et al., 2024) and similarly, preteens are more likely to experience more frequent restrictive mediation compared to older children (Livingstone et al., 2017; Nikken & Jansz, 2014; Symons et al., 2017).

In contrast to television and the internet, relatively little is known about the types of strategies parents use to mediate their children's social media use (Odgers & Jensen, 2020). This is especially true within the preteen age group where, according to socialization theory, the role of parents is particularly influential (Maccoby, 2007), and the risks may be greater due to these children's positivity bias (Boseovski, 2010), trust in information online (Brodsky et al., 2021) and lack of concern and skill in managing their data and privacy online (Gelman et al., 2018; Starks & Reich, 2024).

Recent research by Ho et al. (2020), using focus groups with parents and their 9‐ to 17‐year‐old children, found that in addition to the previously identified strategies of active and restrictive mediation, parents used authoritarian surveillance (knowing the password and accessing their child's account) and non‐intrusive inspection (‘friending’ their child on social media). These focus group responses informed the development of a scale for measuring parental social media mediation, with subscales for each of the four strategies identified in the focus groups. This was validated, and it was found that restrictive mediation was the prevalent approach with preteens (aged 9–13), whereas active mediation was more commonly used for adolescents (aged 14–17). However, Ho et al.'s participants were all from Singapore, and data were collected in 2015. As experiences and opportunities available on social media platforms are continually developing, and as parental control and monitoring have been found to differ across cultures (Lansford, 2022), it is important that this area of research is expanded.

In addition to developing an understanding of parents' mediation approaches, a better understanding of what influences which approaches are used is needed. A recent meta‐analysis (Wang et al., 2023) investigated predictors of parental mediation of children's media use generally and concluded that children's age, together with parental level of involvement and attitude towards media, were the main predictors of the type of mediation strategy used with media type and country of study being important moderators. While studies such as this give us some understanding of the factors that may predict parental mediation of children's social media use, they provide little insight into the bidirectional and multiple factors, including perceptions and experiences, that determine the approaches used.

### Theoretical approach

Parental mediation theory echoes societal expectations that parents should proactively control their child's media use, and strategies used are socially construction (Berger & Luckmann, 1984). Social comparisons (Festinger, 1957) are likely to influence the strategies are used. Furthermore, we realize that some parents may choose not to monitor, limit or discuss media use with their children (Mazmanian & Lanette, 2017). Given the heterogeneity of families and the reciprocal nature of the relationship between parent and child (Bronfenbrenner & Morris, 2007), children's influence on parent's approaches to mediating their preteens social media use should not be underestimated. Indeed, previous research considering parental mediation of internet use found that the mediation strategies employed were co‐constructed by parents and children, and varied depending on the context (Smahelova et al., 2017; Stoilova et al., 2024). This is further support by Clark's (2011) suggestion that to be fit for the digital age, parental mediation theory needs to be extended to consider the emergent strategy of participatory learning that involves parents and children interacting together with and through digital media. Therefore, it is important to gather both the parent's and child's perspectives on what mediation approaches are used and why. Previous research has found that interviewing parents and children together can be a useful methodological approach to elicit effective discussions and co‐construction of ideas (Rose et al., 2022). Moreover, this approach has been found to increase the validity of the data collected and to capture the complexity and subtle nuances of shared experiences (Dale et al., 2021).

A qualitative approach was chosen to ‘enter into the world of participants, to see the world from their perspective, and in doing so make discoveries’ (Corbin & Strauss, 2014, p. 14). Specifically, this research applied the paired‐depth interview approach advocated by Wilson et al. (2016) with parent–child dyads to gather in‐depth insight and rich data to address the research questions of firstly, ‘What strategies are used to mediate social media use among preteens?’ and secondly, ‘What are parents and children's reflections of why different strategies are chosen?’

## METHOD

### Participants

Nine parents (aged 35–53 years; 8 female; M age 44.12 years) and nine preteens (9‐ to 121‐years‐old, M age 10.88 years; three female) participated, resulting in nine parent–child interviews. This sample size is within the range of six to ten interviews suggested by Braun and Clarke (2013) to collect the quality of data required for an in‐depth thematic analysis. Participants were generally white and from middle‐class backgrounds in the Midlands and Northwest of England. Although 8‐year‐old children are commonly included in the age range referred to as preteens, these youngest preteens were not sampled as we wanted all our participants to have sufficient lived experience as a preteen, or parent of a preteen, to draw on for their responses. Participating children attended different schools, all used social media, spending on average almost 15 h a week on social media (range of 3–28 h reported). Eight had at least one social media profile, seven owned a mobile phone, which they used to access social media, and the most popular social media sites were YouTube and Snapchat, followed by TikTok and WhatsApp. Parents had a range of socio‐economic and educational backgrounds; see Table S1 for further details.

Ethical approval was granted by the University of Staffordshire. The study was advertised on the second author's social media account, on a university psychology research participation scheme and by word of mouth. Before the interview, parents and children were fully informed of the details of the study. Parents gave verbal and written consent for their own and their child's participation, and children gave verbal and written assent. Participants were fully debriefed at the end of each interview. No participants withdraw, one child requested that a small part of their interview be removed; this was done.

### Procedure

This study was conducted in accordance with the COREQ guidelines. Interviews were carried out by the second author (a mother with extensive experience of working in schools). The interviews were conducted as part of a final year undergraduate psychology project. Qualitative methodology training has been undertaken. Most of the participants had not met the researcher prior to the interviews taking place, with the exception of one adult participant and two parent–child dyads who were acquainted with the researcher (none were close acquaintances). All participants knew that the researcher was undertaking an undergraduate degree and those acquainted with her knew she had two children approaching preteen age.

Prior to the interview, parents and children independently completed an online questionnaire requesting demographic and social media usage details. No definition of social media was given by the researcher as we wanted to gain insight into the perceptions and experiences of our participants based on their own understanding. The children and their parents were then interviewed together in person, either at the university in a private room or in a mutually convenient quiet setting. All interviews were audio recorded. The semi‐structured interview schedule (see Appendix S1), consisting of an opening script and key questions. This was developed by both researchers; feedback was gained from other qualitative researchers, and the first interview was approached as a pilot, with participants being asked about the clarity and flow of questions. Prompts were used by the researcher to encourage full answers and responses from both the child and their parent. Dyads were first asked about the child's experiences of social media and how these were mediated, followed by questions surrounding the reasons for decisions about mediation approaches.

The potential of a power imbalance and the interview being dominated by one person (Arksey, 1996; Houssart & Evens, 2011) was considered and mitigated by the researcher emphasizing immediately prior to the interview that both perspectives were equally valued and directing questions as necessary to ensure that both voices were heard. Although the power imbalance was a potential risk, it has also been suggested that paired‐depth interviews provide a positive experience for both children and their parents (Rose et al., 2022) serving to minimize future conflict by avoiding tension which can arise when participants are interviewed separately and then want to know what each other have said (Valentine, 1999).

### Analysis

The interviews were recorded (4 h, 48 min, and 9 s) and transcribed verbatim. Participants were given the opportunity to view transcripts; no comments or corrections were received. Two complementary approaches were used to analyse the data: content analysis was used to identify strategies used to mediate social media use among preteens (research question 1) (following Ho et al., 2020) and reflexive thematic analysis (Braun & Clarke, 2006, 2013, 2022) was used to develop themes to reflect parents' and preteens' perceptions of why different strategies were chosen (research question 2). The research was rooted in a critical realist ontological ideology, which considers there to be multiple truths and that knowledge is co‐constructed, with experiences from the outside world only accessible through the lens of perception (Braun & Clarke, 2022). In relation to this, it is important to note that both authors were white middle‐class mothers who had children approaching the preteen age; this gave them an affinity with the participants and may have promoted the sharing of information during the interviews and a sense of having a shared understanding during the analysis.

The content analysis involved both authors reading through the transcripts to gain familiarity with the data. Initially, a deductive approach was used as each interview was scored independently by each author for the presence or absence of *active mediation* (explaining the potential risks and making suggestions about how to manage these), *restrictive mediation* (having rules to restrict time spent or content watched), *authoritarian surveillance* (having full access to their child's account by knowing their password) and *non‐intrusive inspection* (friending their child on social media). The description of each approach provided by Ho et al. (2020) was referred to by the authors during this process. Secondly, an inductive process was used whereby each author independently listed key meaning units (in the form of verbatim quotes) from each interview relating to mediation approaches referred to by either the parent or child, but which were not encompassed by the four approaches identified by Ho et al. These lists were compared and discussed, and a final list of the key meaning units across the nine interviews was established (*N* = 112). Each author independently identified common categories reflecting mediation approaches that were used in addition to those identified by Ho et al. These categories were then discussed and a final list of categories, and descriptions for each was agreed on. Each interview was scored independently by each author for the presence or absence of each category. Any discrepancies in this scoring were discussed and consensus reached.

The reflexive thematic analysis (Braun & Clarke, 2022) was carried out, following the six steps developed by Braun and Clarke (2006, 2013) to address the second research question, focusing on the reflections of why different strategies were chosen. After repeated readings of the transcript, notes of possible codes were made on hard copies of the transcripts. From these, the second author generated the initial themes and the resulting themes, and contributing codes were discussed by both authors. The final themes were agreed upon by both authors, and both were involved in the selection of quotes to illustrate each theme.

## RESULTS

### What strategies are used to mediate social media use among preteens?

Alongside the four strategies reported by Ho et al. (2020) a further 12 strategies were identified during the content analysis. These were subsequently grouped into four broad approaches: ‘active mediation’, ‘restrictions’, ‘surveillance and inspection’ and ‘supporting independence, but monitoring’. The first three of these reflect approaches already identified as being used by parents to mediate children's digital media experiences. However, our findings extend these to social media and provide novel evidence of the active role that children play in mediating their own experiences. Furthermore, the fourth approach (‘supporting independence, but monitoring’) highlights how parents try and support their child's social media use, while giving them independence to make their own decisions. The strategies relating to each approach are detailed in Table 1.

**TABLE 1:** Descriptions of mediation approaches and strategies relating to preteens social media (SM) use.

Approach	Strategy	Definition & example quote	Example quote	Number of interviews^a^
Active mediation	Parent active mediation^b^	Parents advise their children, educate and make suggestions	“I ask my child to tell me [if] something happens on social media, then I give some advice to her and explain the potential risks”	9
	Child‐led active mediation	Child has spoken/would speak to their parent if unsure or concerned about something on SM	“He would come and tell me what's going on”	4
	Balancing out social media with other activities	Valuing other activities, or actively scheduling activities to balance out time spent on SM	“I'm trying to break the week up so that the desire to be on social media won't be quite the same”	5
	Parents as role models	Parents modelling how they would like their child to behave	“I lead by example rather than you can't look at mine, but I can't get yours”	7
Restrictions	Parent restrictive mediation^b^	Parents establish rules to restrict children's behaviours online (rules relate to time, type of activity or content)	“They're not allowed their phones in their bedroom at night…they don't like that, but I think it's a good rule”	9
	Parent technological restrictions	Parent's using technology to restrict apps used and time spent on SM	“No, my dad has to approve with all of my apps”	6
	Child self‐restriction	Child self‐regulated their SM use though restricting their time spent or content	“My phone is mostly on silent and do not disturb so I never really see the messages”	8
Surveillance and inspection	Authoritarian surveillance^b^	Parents log on to their children's SM accounts	“I could go on any time, I know all her log ins and everything”	5
	Parent technological surveillance	Using technology to read messages or interactions	“If anybody tries to make friends with you, now I'll get an email. So I can monitor it that way as well”	4
	Parent device checking	Openly checking the child's device to monitor activity with the child's knowledge	“I sort of look at your phone every week sort of properly”	3
	Using parent's device	Child using parents' device to access SM	“Yeah, uses my phone for making these videos, his shorts so they're all done on my phone”	6
	Parent non‐intrusive inspection^b^	Parents add their children as social media friends in order to browse their profiles and follow their status	“Through Snapchat I can track where they were, when they last used it”	4
	Parent non‐intrusive observation	Being in the same room/walking past whilst the child is on SM with the aim of keep an eye on them	“I just went into his room and I checked what he was doing”	3
	Co‐viewing	Parents sitting together and sharing SM viewing	“When it comes to YouTube, I tend to watch with kids together”	5
Giving independence but monitoring	Parent's taking a step back and trusting	Giving children the freedom to take the next step but being ready to support them	“Try not to do the no thing, try to compromise. If you can get their buy in they're less likely to go against what you said”	4
	Parent waiting and seeing	Taking a step back and waiting to see what happens	“Keep an eye on the mood that's going on to see which way that's going”	5

^a^ This is the number of interviews in which each strategy was present (maximum *n* = 9).

^b^ This strategy is one of the four identified by Ho et al. (2020).

### Reflections of why different strategies are chosen

Choices of mediation strategy were influenced by the desire to maintain a balance (theme 1) between the risks and benefits of social media (theme 1.1) and time spent on social media compared to other activities (theme 1.2). Parents and preteens emphasized the importance of feeling connected (theme 2) and reported that this influenced the type of mediation strategies they used. Parents valued feeling connected to their preteen (theme 2.1) while preteens valued feeling connected to their friends (theme 2.2).

#### Theme 1: Desire to maintain a balance

Parents' and preteens' decisions on how social media use is mediated were influenced by their perceptions of how well preteens maintained a balance between the risks and benefits (1.1) and the time spent on social media compared to other activities (1.2).

##### 1:1 Balancing the risks and benefits of social media use

All parents and preteens discussed the need to mediate the preteen's use of social media to find a balance between reducing the risks and enjoying the benefits of social media. Parents expressed a sense of responsibility in trying to find a balanced approach, which allowed them to support their preteen's developing need for agency and social connection whilst mitigating potential adverse effects of social media. Rather than focusing exclusively on the parents' role, the active role of the preteen was highlighted. In interview two the parent detailed how she considered more invasive mediation approaches as an infringement of her preteen's privacy, while her preteen followed this up with an example of their strategy of completing background checks.

Parent 2 I would prefer to monitor it a bit more, especially now she's getting older, and she's at high school, just to know what she's exposed to. But erm it's a fine line of protection from our side, and you're stepping on… because I think God, if my mum would have read my diary when I was 12 years old, I'd have been grounded forever, and I'd be mortified. So do I want her to have her own things, but at the same time keep her in a safety bubble.

Child 2 Some of my friends from school, if they get a notification saying someone has added them, they'd probably add them back. But I always do like a bit of a background check. Do you know this person? Do I know this person? And if I don't, I'll just block them.

This extract, and many other responses from preteens and parents across all the interviews, suggest that parents recognize their preteen's expectation that they should be trusted more and allowed greater privacy than younger children. Indeed, imposing fewer restrictions would seem an appropriate response as most preteens gave examples of steps that they were taking to mediate their own social media use. It is likely that these mature actions were been recognized by the parent and contribute to them taking a less restrictive approach to mediation. Even when talking about things that have scared them, preteens demonstrated their maturity and own learning. For example, child 9 shared that ‘The thing that scared me the most is when I *thought* my gaming account got hacked, but it was just a bug’. Many parents also spoke about accepting their children would have some negative online experiences but wanting to keep these small so that they could be a learning experience. For example, in interview 9 the parent said You can't shield them from all the negativity from out there it is having that in small doses so that they then sort of grow with themselves to know how to deal with that.

In summary, this subtheme relates to parents AND preteens mediating social media use in a way that respects the preteens' increasing independence and maturity with the shared goal of balancing potential risks and benefits.

##### 1:2 Finding the right balance in time spent on social media

Parents expressed concern when they perceived time spent on social media to be excessive or there to be an imbalance in the time their preteen spent on social media compared to other ‘real‐world’ hobbies and interests. All parents interviewed valued other activities to ‘balance out’ their preteens' use of social media. Some parents talked of their motivation and effort to actively encourage their preteen to take up different activities to ‘distract’ them from using social media, such as in this extract.

Parent 4 She does this video editing…I'm trying to hopefully get her enrolled in a summer school, working with visual artists…she's doing the drama…she's at a youth club I make her go to…she does stage. Because there's lots, I'm trying to break the week up so that the desire to be on social media won't be quite the same.

This parent then goes on to talk about how children used to spend more time outside meeting up with friends, but how now staying in, and using screens, has become ‘the safer option in a way’. At this point the preteen enters back into the conversation with their view on the benefit of social media for connecting with their friends, seemingly trying to explain to their parent that now they can connect with their friends without the need to leave the house: ‘I thought it was good because I could just sit in the comfort of my own home and just talk to my friends and stuff and be with them technically’ (Child 4). These extracts reflect others in the data set in which it was notable that the goal of maintaining a balance between time on social media and other activities was a goal that was often introduced by parents.

Children, and indeed their parents, referred to the extent to which the amount of time spent on social media varies across families. In the extract below, the child is comparing their social media use to that of others and recognizing the need for a balance. The parent follows this up by talking about different mediation approaches.

Child 9 I mean, it's like four of my friends, just, the parents don't care. And they're just on it for like, 24 h straight. But then some of my friends have, like, really strict parents are only allowed on for like, 2 h a day. So, it's more like, I'm kind of in the middle, which I like, I'm not spending too long on it. But I'm not on it for like, too little. So, it's that balance.

Parent 9 I think for us, it's, if you're strict, then it's like becomes like the forbidden thing doesn't it that they want to do it more, and I don't want him going behind my back and doing things that I don't want him to do because he thinks that that's it. I think we've been a bit more relaxed with him so that he can then grow independently, and decide for himself what he thinks is enough and make them hopefully for us make the right choices which up to now you have done.

Throughout this subtheme, the concept of there being an appropriate balance in the amount of time spent was shared by both parents and their preteens. Comparisons were made by both preteens and their parents to variations in how parents controlled, or didn't control their preteen's screentime. This was much more nuanced than parents thinking screen time should be more restricted and preteens thinking it should not be restricted at all, instead there seemed to be clear agreement that a balance was required, as demonstrated by parent and child 9 in the extract above. Furthermore, there was evidence throughout the interviews of children placing some restrictions on their own use. For example, Child 5 says, ‘My mum doesn't make me feel restricted, but I feel like I make myself be restricted more. So, I'm restricting myself more than my parents restrict me’.

#### Theme 2: Desire to feel connected

Parents and preteens reported that feeling connected (or not) impacted mediation approaches used. While parents valued feeling connected to their preteen (2.1), preteens valued feeling connected to their friends (2.2).

##### 2:1 Parents value feeling connected to their preteen

Throughout the interviews, parents consistently referred to wanting to be aware of their preteens' social media activity and to be able to have open and honest communication with them. Throughout the interviews, parents expressed how they felt more relaxed about their preteens' social media use when they felt that they could trust them, as demonstrated in the following exchange.

Parent 5 He's actually so well behaved that if you say don't use it, he won't use it. He's not one that's gonna go behind your back or anything. Yeah, I think it's good for them to use it so that when they get older, they're not going to have hangups and issues, because anything that goes on now, we're there to help him deal with…

Child 5 Parents may not always be right, but they usually do have a point. And they do have logic behind what they're saying. Maybe you can find a workaround together.

However, when the same parent was asked how they would react if their preteen became more secretive, this parent explained that their strategy would become quite different as they would ‘Talk. Ask. Keep asking. He knows jolly well I can get whatever I want, when I want and how I want from his phone…he knows that I can access anything’. For this parent, a lack of transparency around their preteens' social media activity would result in them choosing intrusive inspection as a mediation strategy, demonstrating that parents' feelings of connection to their child directly influence their choice of mediation strategies. In this instance, the preteen seemed to see this a threat that will act as a deterrent, saying: ‘Yeah, so it's not worth it’.

Evidence of the strategies used to achieve trust between parent and child, including recognizing the child's increasing maturity and need for independence, is also referred to in the extracts within Theme 1.1. This need to trust and feel connected to their preteen was emphasized by parents throughout the interviews and appeared to be something that was respected by preteens who recognized it as important to their parents.

##### 2:2 Preteens value feeling connected with friends

During the interviews, preteens discussed how they valued the opportunity to connect with their friends on social media. For example, the preteen in interview one explains that it enables him to feel more connected to friends, particularly during the school holidays.

Probably talking to your friends and that like now we've got 2 weeks or a month off or something like that at most of those days you're either doing stuff or like you can't really chat to anyone so you can like check up on them.

However, the parent of this preteen also talked about their preteen putting their phone on ‘do not disturb’ when they got too many notifications from a group chat, saying ‘He'll get to a point where he's like I don't want to speak to anyone, and you'll put your phone on do not disturb don't you?’. Similarly, another preteen talks about a group they are part of on social media saying ‘Not bothered. I take a quick look when it says that there's like 100 notifications’ (Child 2). Both these extracts seem to suggest that preteens can self‐regulate their social media use and ignore notifications. However, this contrast the way that many preteens talked about needing to check and respond to messages from close friends. For example, the preteen in interview 3 explains ‘when we found out we could chat with each other, we started really started to like, get excited to go home from school and we used to like come on straight away. To see if they were on an app’. The parent in this interview also recognizes the importance of social media for their preteen feeling connected with their friends and perceives that placing excessive restrictions would likely result in their preteen experiencing social isolation.

Parent 3 Now everybody knows where everybody is all the time, especially with things like Snapchat, there's a map with them all on so they can look at where they all are. And it's really unhealthy. However, I look at it the other way and think well, that is it's hard for me to understand that is the way that they communicate, and it's not going anywhere, you can keep them off it but then they're just actually isolated from you know, those social circles. So yeah, it that's their social circle. So that's how they, that's how they communicate. And if you exclude them from that, then you're also almost sort of giving them you know, making them look like a social pariah because they're not involved in it.

Within this theme feeling connected to friends was not necessarily a joint goal as it was emphasized more by preteens than their parents. However, parents understood their preteens need to feel connected and this influenced them to choose less restrictive mediation strategies.

## DISCUSSION

The current research aimed to use paired‐depth interviews with parent–child dyads to address the research questions of two research questions: (1) ‘What strategies are used to mediate social media use among preteens?’ and (2) ‘What are parents' and their preteens' reflections of why different strategies are chosen?’. In response to the first question, 16 distinct mediation strategies were identified from the nine parent–child interviews using content analysis. This extends previous understanding beyond the four strategies identified by Ho et al. (2020) reflecting the diversity of approaches experienced by preteens. For example, Ho et al.'s approach of active mediation focused only on parents speaking to their children; however, our evidence suggests that parents use more varied strategies to try and educate their children and suggests ways for them to stay safe, including modelling the behaviour they would like their child to practice and actively encouraging other activities to balance out their child's social media use. Furthermore, our data suggests that children have an active role in mediating their social media use by initiating conversations with parents. Similarly, restrictive mediation strategies are broader than parents' making rules and include using technology to apply the restrictions and children restricting themselves.

It was beyond the scope of our sample to investigate potential patterns in the use of strategies in relation to background factors such as children's age or gender, parents' educational background or socio‐economic background. What our findings provide is the evidence and rationale for future research to consider mediation approaches beyond the four identified by Ho et al. (2020).

The importance of open communication and collaborative approaches to mediation was also emphasized within the four themes generated from the interviews in response to our second research question: ‘What are parents' and their preteens' reflections of why different strategies are chosen?’ The first theme focused on using mediation approaches *to maintain a balance* between risks and benefits of social media use (theme 1.1) and time spent on social media (theme 1.2) The second theme reflected how the goals of feeling connected, specifically parents feeling connected to their preteen (theme 2.1) and preteens feeling connected to their friends, were related to selecting mediation approaches.

Within theme 1.1 (Balancing the risks and benefits of social media use) it was clear that although preteens may still be developing their ability to think critically and to manage their online behaviours (Gelman et al., 2018; Starks & Reich, 2024) those who took part in our interviews demonstrated maturity and an understanding of online risks and need to mediate online behaviours. This echoes previous findings using paired‐depth interview with parents and their preteens (10‐ to 11‐year‐olds) which focused on mobile phone use, in which children surprised their parents with knowledge of risks and their mature attitudes (Rose et al., 2022). Parents were aware of preteens' potential vulnerability and felt a responsibility to protect them, but also wanted to give them independence and some online freedoms. One reason given within our data for giving some online freedom was that parents seemed to value preteens experiencing some risk on social media to enable them to learn how to deal with issues when they arise, and thereby potentially developing their ability to recognize, manage and recover from online risks (Livingstone et al., 2017).

In contrast to Theme 1.1 being a shared goal, Theme 1.2, balancing time spent on social media compared to other activities, was a goal expressed by parents, and respected, but maybe not shared, by their preteens. This concern about time spent on social media from parents reflects previous research findings that parents associated the internet with an impoverishment of their children's lifestyle, with many preferring their children to engage in offline activities (Hernan‐Garcia et al., 2021). However, ideas about what, if any, is the appropriate amount of time to spend on social media and what is a ‘good’ balance are clearly a social construction, with both preteens and their parents making social comparisons as they try to justify their ‘balance’.

Theme 2.1, parents valuing feeling connected to their preteen, reflected the desire of parents to have open and trusting relationships with their preteens. This echoes research into technology more broadly, where parents of slightly younger children (7‐ to 8‐year‐olds) have expressed their appreciation when their children communicated with them about their technology usage (Smahelova et al., 2017) and conclusions from a rapid evidence review of why parents use digital parenting controls, which emphasized the benefits of joint rule setting, child involvement in decision making and open communication (Stoilova et al., 2024).

However, what was also clear from our data was that if a lack of trust developed for any reason, parents would quickly adapt their style and impose a greater number of restrictions and closer monitoring. This corroborates research which suggests that if parents believe their child is more vulnerable to online risks, which could include not being open and talking to their parent about an online experience they have had, they will increase the restrictions (Nikken & Jansz, 2014). Conversely, parents recognized the importance of social media for their preteen feeling socially connected to their friends (theme 2.2) and felt that placing harsh restrictions could leave their preteen socially isolated and lacking access to a potential source of social support, as documented by Cole et al. (2017).

The opportunity to recognize children's agency and active role in decisions about social media mediation are clearly evidenced throughout all themes. Parents valued their preteens' maturity and own mediation strategies and these gained parents trust, resulting in parents placing fewer restrictions and monitoring their preteen's usage less. This reflects recent evidence that parents who value trust and autonomy tend to engage in less parental mediation (Young et al., 2023) and that when children act more maturely parents tend to impose fewer restrictions on their media use (Ho et al., 2020; Livingstone et al., 2017; Wang et al., 2023). Furthermore, it aligns with the strategies of parents ‘taking a step back and trusting’ and ‘waiting and seeing’ which were identified within our own content analysis of mediation strategies.

Previous research has focused on the extent to which parents' experiences and demographics and child demographics predict choice of strategies used by parents to mediate children's social media use (Wang et al., 2023). However, our findings provide evidence that this is an oversimplistic approach, as strategy choice is the result of a dynamic and continually evolving context which reflects both parents' and children's perceptions and desires. Furthermore, throughout the interviews, there was extensive evidence that preteens experience a wide range of approaches applied in combination. This challenges any suggestion that parents apply only one mediation strategy and has implications for future research investigating the effectiveness of different strategies, as assessing the effectiveness of one approach in isolation may be too reductionist. Instead, the combination of approaches used must be considered in relation to one another, children's social media experiences and broader development. In support of using a combined approach, a recent review into the extent to which technology‐based parental controls are effective for keeping children safe online concluded that, as a standalone strategy, parental controls were not effective, but that when parents integrate the controls into an approach emphasizing open communication, joint rule setting and child involvement in decision making, then the best outcomes for the child occur (Stoilova et al., 2024).

### Limitations

Although children attended different schools (urban and semi‐rural) and parents had a range of socio‐economic and educational backgrounds, it should also be noted that participants are likely to have been a relatively homogeneous group of parent–child dyads who had the time and willingness to take part in this research. Specifically, parents and children who already engaged in open discussions about social media use may have been more likely to participate. Therefore, the views presented may be most representative of parents who take a more active mediation approach where discussion with the child is valued. Similarly, parents who had a good relationship with their child within the context of social media may have been more likely to volunteer, as those parents experiencing conflict may have been less likely to volunteer to take part. This may have resulted in the impression that mediation approaches were amicably co‐constructed; however, we recognize that this will not always be the case. Furthermore, although steps were taken to address potential power imbalance between it is possible that preteens may have chosen to express views which they believed adults would approve of. This could have further added to the impression that the preteen and parent had shared values and that mediation approaches were amicably co‐constructed. To address this, future research could actively try to recruit a more diverse sample and could also collect data from preteens through peer‐facilitated focus groups to reduce potential power imbalance. Furthermore, due to the small scale and qualitative design of this study, no inferences can be drawn about the prevalence or frequency of the observed findings that go beyond our current sample.

### Future directions

Future research should provide opportunities for parents and children to report a broad range of mediation approaches, rather than assuming that parents have a typical or single approach. This is likely to be an oversimplistic approach, as strategy choice is the result of a dynamic and continually evolving context in which the parent and child both have active roles. Consequently, future research and policies must consider the role of the child in actively co‐constructing the mediation of their own social media experiences.

## CONCLUSIONS

Our findings challenge research suggesting that parents have a typical mediation approach, suggesting instead that parents use multiple approaches interchangeably depending on context. Furthermore, the role of children in co‐constructing the mediation strategies and taking an active role in applying them to their own social media use must be considered. This has implications for future policies, interventions and research into the effectiveness of different mediation approaches.

## AUTHOR CONTRIBUTIONS

**Sarah E. Rose:** Conceptualization; methodology; data curation; supervision; formal analysis; validation; writing – original draft; writing – review and editing. **Louise R. Middling:** Conceptualization; methodology; data curation; investigation; validation; formal analysis; project administration; writing – review and editing; writing – original draft.

## FUNDING INFORMATION

This research received no specific grant from any funding agency in the public, commercial or not‐for‐profit sectors.

## CONFLICT OF INTEREST STATEMENT

The authors do not have any conflicts of interest to report.

## Supporting information


Appendix S1

Table S1

## Data Availability

Due to the nature of this research, participants of this study did not agree to have their data shared publicly, so supporting data is not available.
