# Supplementary material for: Preteens social media use: Parents' and children's perceptions of what mediation approaches are used and why
Source: Br J Dev Psychol. 2025 Feb 25;43(3):771–86. doi: 10.1111/bjdp.12552 (PMC12351206; doi:10.1111/bjdp.12552)
Supplement: Supplementary file 1 — Appendix S1 [file BJDP-43-771-s002.docx]

**Interview script and schedule**

Interview schedule for parents and children

**Welcome script**

First of all, I’d like to thank you both for agreeing to take part and also you welcome to the interview. I’d like to discuss the aim of our meeting today which is to get an idea of both of your views and experiences of your (gesture to child) experiences of using social media. There are no right or wrong answers, and I would like to encourage you to be as open and honest as possible to get a true picture of each of your experiences. As with all perspectives, it may be that you have similar or very different views and perceptions to each other, and I’d like to reassure you that this is completely natural and anything that we discuss will be strictly confidential and anonymous. If at any point you feel uncomfortable, would like to take a break or decide to end your interview you can do so at any time without explanation. If you decide to withdraw, I will provide you with a copy of the debriefing sheet, which contains information about sources of support you can access if there is anything you wish to talk about in confidence. You can also withdraw your data from the study after you have finished participating up to two weeks after the date of your interview after which withdrawal of your data will no longer be possible as the data will already have been processed. To withdraw from the study, please email me providing the pseudonym that you have given. Can I just check that on this basis you are happy to proceed? Before we start do you have any questions?

**Questions**

1. (To child) So in the quiz you completed, you said that you use/do not use social media. Can you tell me a little more about that? / Why do you think you haven’t used social media so far? Why did you choose the sites you did?
2. (To Parent) Can I ask you how you have found the experience so far?
3. If I can ask both of you here, was it an easy decision for you/your child to start using social media? What made you decide to use/not use SNS? Is there anything that you would change or do differently? What did you have to think about before using it?
4. Do you/would you have any rules for social media use? How did you/would come to these? Have these/would these been/be changed along the way? Are they working for both of you?
5. Would you like to see any changes in how you/your child (name) uses social media/ Would you like to start using social media? Why?
6. What is your biggest risk and biggest benefit of using/your child using social media?
7. How do you see your SNS use changing in the next two years? What would that look like? Would the agreement between you change do you think? How would you reach an agreement?
8. Is your social media use/approach to rules similar to other children in your class/ other friends or parents? What made you decide that this was the best way forward?
9. Is there anything else that you would like to add?

This is now the end of the interview. Thank you both very much for your time!
