# Supplementary material for: Preteens social media use: Parents' and children's perceptions of what mediation approaches are used and why
Source: Br J Dev Psychol. 2025 Feb 25;43(3):771–86. doi: 10.1111/bjdp.12552 (PMC12351206; doi:10.1111/bjdp.12552)
Supplement: Supplementary file 2 — Table S1 [file BJDP-43-771-s001.docx]

**Table 1**

*Participant demographics and data surrounding the usage of Social Media (SM)*

| Paired Dyads | Gender: Age of parent, child | Gender: Age of sibling(s) | Education &/household income  (in 1,000s) | Parental use of SM | Child Age &/App first used | Child has an SM account? &/Apps used * | Child’s hrs per week on SM  Parent/  Child |
| --- | --- | --- | --- | --- | --- | --- | --- |
| 1 | F38, M11 | M14 | Degree  £60-99 | Average | 5 YT | Yes  SN,TT,WA,YT | 14/14 |
| 2 | F34, F12 | M9,M2 | Some university/ £20-40 | Below Average | 9 SN | Yes  SN, IN | 12/16 |
| 3 | F43, M9 | F14 | Vocational/ £60-99 | Above Average | 6 YT | No  YT | - /7 |
| 4 | F48, F11 | F16 | Degree  £60-99 | Average | 9 WA | Yes  BR,SN,TT,WA,  YT | 15+/28 |
| 5 | F50, M10 | - | Post Grad  £60-99 | Below Average | 7 SN | Yes  SN, WA, YT | 15/14 |
| 6 | F53,  F12 | F10 | Vocational  £20-40 | Average | 7 YT | Yes  IN,SN,TT, YT | 3/3 |
| 7 | M40,  M10 | M7 | Secondary school  £100 + | Above Average | 8 YT | Yes  YT | 10/10 |
| 8 | F35,  M12 | M15 | Degree  £20-40 | Average | 6 YT | Yes  FB,SN,YT | 7/15+ |
| 9 | F46,  M12 | M8 | Post Grad  £60-99 | Average | 10 YT | Yes  SN, TT, TW, TWI, WA, YT | 25/25 |

*BR= Be Real, FB= Facebook, IN=Instagram, SN=Snapchat, TT=Twitter, TWI=Twitch, WA=WhatsApp, YT=YouTube
